# Unilateral Hyperkinetic Choreiform Movements due to Calcification of the Putamen and Caudate from an Underlying Developmental Venous Anomaly

**DOI:** 10.7759/cureus.3990

**Published:** 2019-01-31

**Authors:** Ramsey A Falconer, Tulsi Shah, Anne Giles, Mahesh Shenai, Sean Rogers

**Affiliations:** 1 Neurology, Inova Health System, Falls Church, USA; 2 Neurology, Virginia Commonwealth University School of Medicine, Fairfax, USA; 3 Neurosurgery, Inova Health System, Falls Church, USA

**Keywords:** hyperkinetic movement disorders, hemibalism, chorea, basal ganglia injury, developmental venous anomaly, unilateral movement disorder, unilateral chorea

## Abstract

This index case report describes a patient who presented with unilateral hyperkinetic choreiform movements of the left neck, arm, and leg caused by right-sided putamen and caudate calcification as the product of an underlying developmental venous anomaly (DVA). No underlying metabolic disorder or other calcium-related disorder was present.

Calcification of the putamen and caudate has been described in relation to an underlying DVA which results in localized venous hypertension and other changes, and tends to spare the anterior limb of the internal capsule.

This resulting unilateral choreiform movement disorder has not been described in the literature and represents the need for greater recognition of this entity in the differential for lateralizing hyperkinetic disorders.

## Introduction

The category of hyperkinetic movement disorders includes several conditions whose hallmark symptoms involve movements that are large in amplitude, fluctuating in severity, irregular and abrupt. Termed chorea, these movements can be explained by a broad differential involving basal ganglia pathology [1]. The most well known is Huntington’s disease, whose neuropathology is theorized to be a result of neuron loss in the striatal part of the basal ganglia, specifically the caudate and putamen [2-3]. Similar in symptom presentation but differing in pathology, ischemic stroke to the structures of the striatum has been shown to cause abnormal hyperkinetic movements contralateral to the brain hemisphere affected [4-5].

The caudate and putamen are the theorized origin of hyperkinetic disorders due to their role as the afferent pathway of the basal ganglia, and major source of inhibition of the globus pallidus externa (GPe). When injured or degenerated, the caudate and putamen are no longer able to adequately transport the major inhibitory transmitter gamma-aminobutyric acid (GABA) to the globus pallidus, thereby relieving it and other structures of their inhibitory effect on the thalamus, resulting in hyperkinetic movements [6].

Unilateral pathology in the basal ganglia structures of the striatum are rare, and described in the literature mainly as the result of stroke (hemorrhagic or ischemic) [4-5]. Beyond stroke, unilateral calcification of the putamen and caudate as a product of a developmental venous anomaly (DVA) have been described through a small number of case reports, but none resulting in abnormal movements. 

DVAs are venous malformations that drain normal brain tissue with an abnormal, centripetally converging network of veins that then converges on a large trunk which continues to normal vasculature [7]. Described as ‘spokes on a wheel’, these vascular anomalies are considered the most frequently encountered cerebral vascular malformation, present in around 3% of patients [8-9]. These malformations are usually incidental findings on brain imaging and almost always result in a benign course [10]. It is theorized that due to the vascular dynamics at play, localized venous hypertension is present in the territory drained by these malformations, which can result in gliosis, encephalomalacia as well as parenchymal calcification [7,10-12].

Through this case report, we describe an interesting patient who presented with severe unilateral choreiform movement and was then found to have corresponding contralateral putamen and caudate calcification on computed tomography (CT) of the head, with anatomically adjacent DVA seen on magnetic resonance imaging (MRI) of the brain.

The association between unilateral putamen and caudate calcification in association with a DVA has been described only a number of times in the literature: six cases [11], two cases [13], and one case [10]. These all interestingly spared the anterior limb of the internal capsule. None of these prior cases reported the presence of hyperkinetic movement symptoms.

## Case presentation

Our patient is a 67-year-old male with a history of hypertension, type 2 diabetes, and hyperlipidemia, who went through the Inova Movement Disorders Program due to a history of fluctuating, progressing uncontrollable movements of the left side of his body. He reports these movements had been present for around 10 years, worsening over time.

At baseline, he always felt that his left arm and leg were moving, at times exhibiting large amplitude movements which could knock things down or affect his walking, making him fall. He described it as abnormal, uncontrollable movements that made his arm ‘reach around or fling out’ His leg was also described to ‘dance on its own.’ While at baseline, these were present but minimal stress or anxiety would exacerbate the movements. There were no movement issues on his right side, nor any facial movements noted.

His exam showed episodic, mild with some interspersed moderate-amplitude quick movements of the left hand, forearm, proximal arm muscles, as well as movement of the left leg. These were not suppressible, brought out more by distraction or with anxiety, such as discussing an MRI scan as he was claustrophobic. He also demonstrated some lateralizing movements of the head to the left, but no facial or tongue movements. His right side showed no such signs, and his neurological exam was otherwise unremarkable.

**Figure 1:**
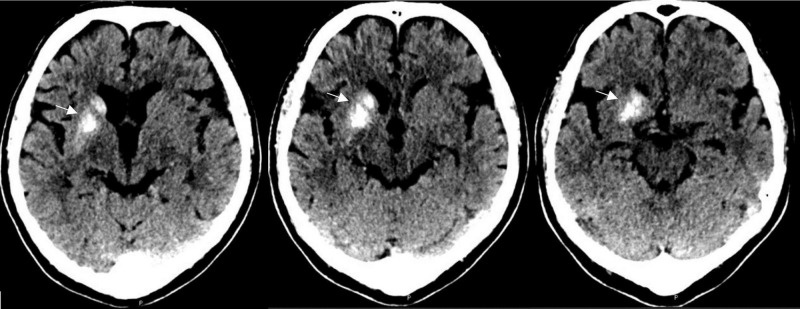
Computed tomography (CT) of the head without contrast showing unilateral putamen and caudate calcification sparing the anterior limb of the internal capsule

**Figure 2:**
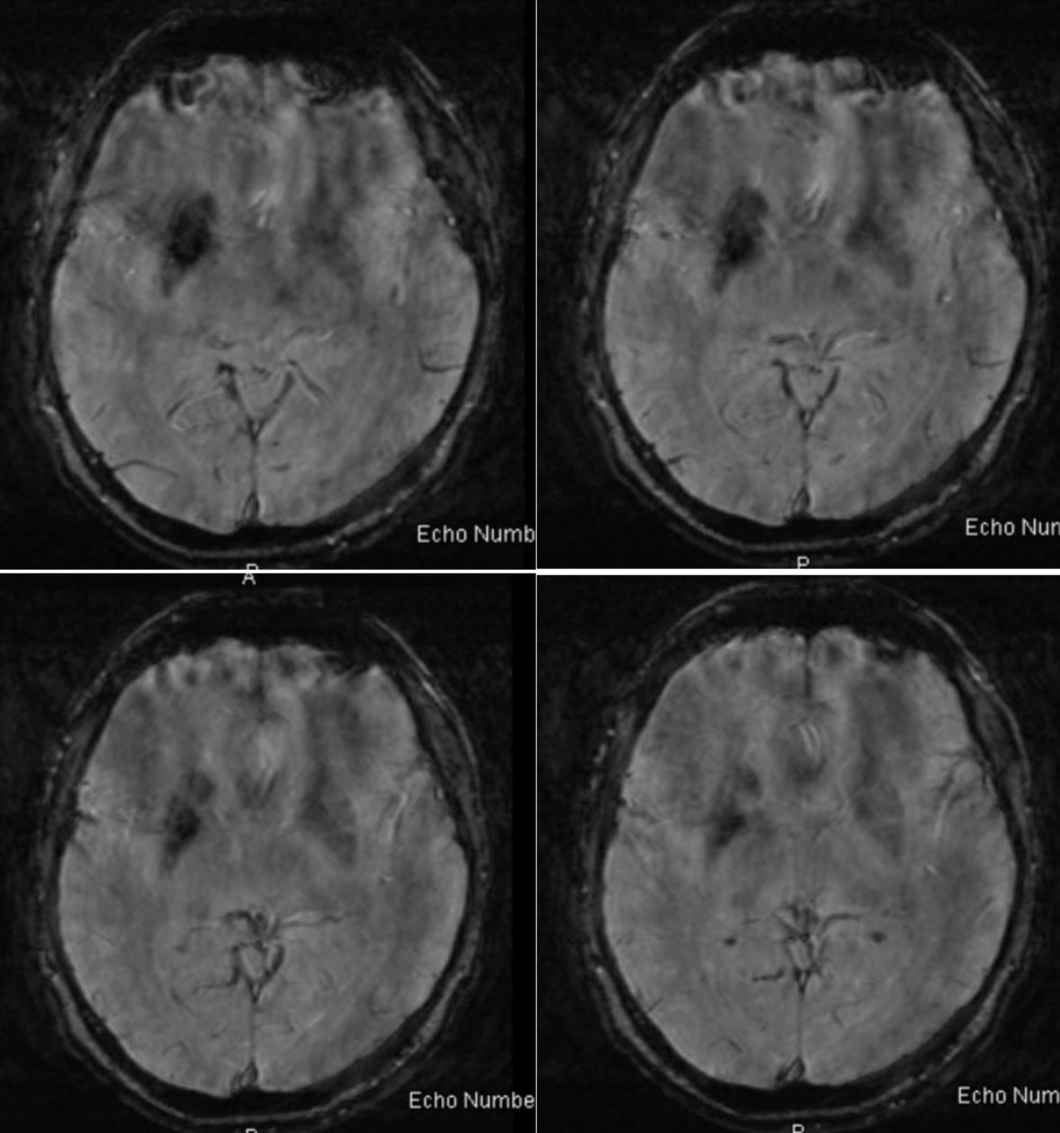
Gradient echo (GRE) imaging showing hypointensity in the area of caudate and putamen reflecting calcification

**Figure 3:**
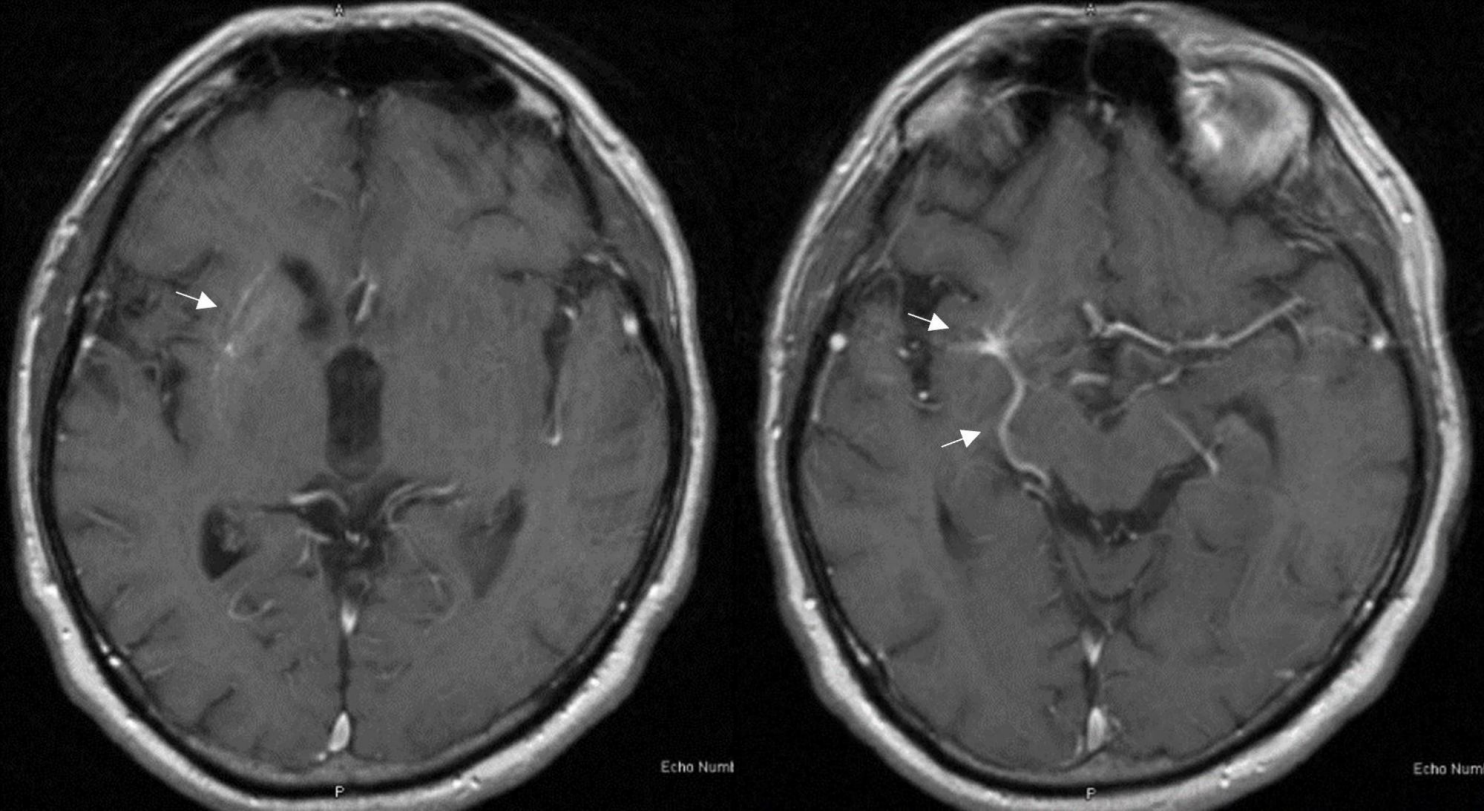
T1 magnetic resonance imaging (MRI) of the brain with contrast showing right-sided developmental venous anomaly (DVA)

He was sent for a CT scan of the head initially due to a fear of MRI scanners, and was found to have asymmetric calcification of the right caudate nucleus and anterior putamen (Figure 1). The calcification spared the anterior limb of the internal capsule. Laboratory work for parenchymal calcification was then sent and was normal. The MRI of the brain with contrast demonstrated both hypointense gradient echo (GRE) signal in the right caudate nucleus and putamen, as well as an underlying DVA (Figures 2-3).

Given the lateralizing of his deficits and correlation with opposite hemispheric parenchymal calcification, he was diagnosed with unilateral hyperkinetic movements as a product of putamen and caudate calcification from an underlying DVA. Consideration was made for focal hemorrhage, but presentation and imaging were not thought to reflect this pathology. He was then treated with 0.25 mg clonazepam 2x daily with significant improvement in his hyperkinetic movements.

## Discussion

The presentation of unilateral choreiform movements has a limited differential diagnosis outside of the focal injury to the putamen and caudate due to stroke [4-5]. Being the pathway which results in thalamic inhibition, any disruption or injury to the putamen and caudate has the potential to release that inhibition and result in hyperkinetic movements. When injured or degenerated, the caudate and putamen are no longer able to adequately transport the major inhibitory transmitter GABA to the globus pallidus, thereby relieving it and other structures of their inhibitory effect on the thalamus, resulting in hyperkinetic movements [6]. As DVAs are common vascular malformations, and in a rare case has been shown to cause unilateral calcification of the caudate and anterior putamen due to presumed venous hypertension in the territory drained by the DVA, it therefore makes theoretical sense that the mineralization of the putamen and caudate as well as localized venous hypertension could also impede this pathway [7,10-12].

## Conclusions

This index case report describes a patient who presented with unilateral hyperkinetic choreiform movements of the left neck, arm, and leg caused by right-sided putamen and caudate calcification as the product of an underlying DVA. We believe that this case serves as evidence that unilateral striatal calcification due to an underlying DVA should be part of the differential of unilateral hyperkinetic choreiform movements.

## References

[1] Reiner A, Dragatsis I, Dietrich P (2011). Genetics and neuopathology of Huntington’s disease. Int Rev Neurobiol.

[2] Albin RL, Young AB, Penney JB (1989). The functional anatomy of basal ganglia disorders. Trends Neurosci.

[3] Chung SJ, Im JH, Lee MC, Kim JS (2004). Hemichorea after stroke: clinical-radiological correlation. J Neurol.

[4] Whittier JR (1947). Ballism and the subthalamic nucleus (nucleus hypothalamicus; corpus luysi): review of the literature and study of 30 cases. Arch Neur Psych.

[5] Bhidayasiri R, Truong DD (2004). Chorea and related disorders. Postgrad Med J.

[6] Park J (2016). Movement disorders following cerebrovascular lesion in the basal ganglia circuit. J Mov Disord.

[7] Dillon WP (1997). Cryptic vascular malformations: controversies in terminology, diagnosis, pathophysiology, and treatment. Am J Neuroradiol.

[8] Garner TB, Curling OD Jr, Kelly DL Jr, Laster DW (1991). The natural history of intracranial venous angiomas. J Neurosurg.

[9] Aoki R, Srivatanakul K (2016). Developmental venous anomaly: benign or not benign. Neurol Med Chir.

[10] Ruiz DSM, Delavelle J, Yilmaz H (2007). Parenchymal abnormalities associated with developmental venous anomalies. Neuroradiology.

[11] Dehkharghani S, Dillon WP, Bryant SO, Fischbein NJ (2010). Unilateral calcification of the caudate and putamen: association with underlying developmental venous anomaly. Am J Neuroradiol.

[12] McCormick WF (1966). The pathology of vascular (“arteriovenous”) malformations. J Neurosurg.

[13] Sarp AF, Batki O, Gelal MF (2015). Developmental venous anomaly with asymmetrical basal ganglia calcification: two case reports and review of the literature. Iran J Radiol.

